# SKF96365 Inhibits Tumor Proliferation by Inducing Apoptosis and Autophagy in Human Esophageal Squamous Cell Carcinoma

**DOI:** 10.1155/2024/4501154

**Published:** 2024-08-13

**Authors:** Jiaxin Zhang, Huiqiong Han, Yihan Liu, Jiayao Xu, Daidi Zhang, Wenjia Wang, Yaping Gao, Zhengrui Li, Yanru Qin

**Affiliations:** ^1^ Department of Oncology The First Affiliated Hospital of Zhengzhou University, Zhengzhou 450052, China; ^2^ Department of Oral and Maxillofacial-Head and Neck Oncology Shanghai Ninth People's Hospital Shanghai Jiao Tong University School of Medicine, Shanghai 200011, China

**Keywords:** apoptosis, autophagy, calcium channel, esophageal squamous cell carcinoma, SKF96365, TRPC1

## Abstract

Calcium channel blockers are emerging as a new generation of attractive anticancer drugs. SKF96365, originally thought to be a store-operated calcium entry (SOCE) inhibitor, is now often used as a TRPC channel blocker and is widely used in medical diagnostics. SKF96365 has shown antitumor effects on a variety of cancer cell lines. The objective of this study was to investigate the anticancer effect of SKF96365 on esophageal cancer in vivo and in vitro. Cell Counting Kit-8 (CCK-8) and colony formation were used to test the proliferation inhibition of SKF96365 on cell lines. Western blot and terminal deoxynucleotidyl transferase dUTP nick end labeling (TUNEL) staining were used to detect cell apoptosis rates. In addition, we demonstrated the antitumor effect of SKF96365 in vivo in xenografted mice. As a result, SKF96365 significantly inhibited the proliferation of K510, K30, and EC9706 in vitro. SKF96365 induces apoptosis in three cell lines through the poly(adenosine diphosphate–ribose) polymerase (PARP), caspase-9, and BCL-2 pathways in a dose-dependent and time-dependent manner. Moreover, SKF96365 treatment also induced apoptosis and inhibited tumor growth in nude mice. The calcium channel TRPC1 was significantly downregulated by SKF96365. Autophagy was also induced during the treatment of SKF96365. In summary, SKF96365 induces apoptosis (PARP, caspase-9, and BCL-2) and autophagy (LC3-A/B) by inhibiting TRPC1 in esophageal cancer cells, thereby inhibiting tumor growth.

## 1. Introduction

Esophageal cancer is the seventh-most common tumor in the world. In our country, esophageal squamous cell carcinoma (ESCC) is the most common histological classification, followed by adenocarcinoma. Despite improvements in treatment outcomes over the past decade, the 5-year survival rate for patients with esophageal cancer remains below 20% in most parts of the world [1]. Currently, the treatment options for esophageal cancer are limited to surgery, chemoradiotherapy, immunotherapy, targeted therapy, and combination therapy. The occurrence of multiple adverse reactions and acquired drug resistance are new clinical challenges [2]. Finding novel therapeutic drugs is the cornerstone of exploring higher value therapeutic modalities.

In recent years, the identification of novel biomarkers in various diseases has made significant advances, deepening our understanding of the diseases and sharing more mechanistic insights into their pathogenesis [3]. An emerging and exciting area of cancer research is searching for calcium signaling pathways in cancer cells. Calcium channels are closely related to apoptosis and autophagy. Previous research has established that the disruption of physiological Ca^+^ signaling can induce calcium oscillations, consequently generating signals to regulate cell functions such as apoptosis, autophagy, differentiation, and proliferation or invasiveness [4–7]. SKF96365, a calcium antagonist, has previously been widely used to explore the pathophysiological function of store-operated calcium entry (SOCE) in nonexcitatory cells [8]. Currently, it is often used in medical research as a TRPC channel blocker. SKF96365 has shown potential cytotoxicity in several human cancers. For example, Jing et al. reported that SKF96365-induced G2/M cell arrest and apoptosis accompanied autophagy in colorectal cancer cells, providing a promising strategy in combination with hydroxychloroquine [9]. Bai, Chen, and Zheng demonstrated that DNA synthesis can be inhibited by SKF96365 treatment in cervical cancer [10]. Intraperitoneal injection of SKF96365 distinctly restrained the growth of nasopharyngeal carcinoma, gastric carcinoma, and breast carcinoma in xenografted nude mice [11–13]. However, the mechanism by which SKF96365 inhibits the growth of esophageal cancer remains unclear.

In this study, we mainly focused on the inhibitory effect of SKF96365 on esophageal cancer in vivo and in vitro. Mechanically, SKF96365 inhibits the expression of TRPC1; induces apoptosis via poly(adenosine diphosphate–ribose) polymerase (PARP), caspase-9, and BCL-2; and induces autophagy via LC3-A/B in K510, K30, and EC9706 cell lines. Additionally, we also confirmed this apoptosis mechanism in nude mice, and SKF96365 showed a satisfactory antitumor effect, which has critical guiding significance for future treatment of esophageal cancer.

## 2. Materials and Methods

### 2.1. Cell Line Culture

Human ESCC cell lines used in this study included KYSE-510 (RPMI-1640), KYSE-30 (RPMI-1640), and EC9706 (RPMI-1640), which were obtained from the Cell Bank of Type Culture Collection of the Chinese Academy of Sciences. All culture media were obtained from Gibco BRL Life Technologies. All cells were cultured in a medium supplemented with 10% FBS and 1% penicillin–streptomycin (all from Gibco; Thermo Fisher Scientific Inc.) at 37°C in humidified air with 5% CO_2_.

### 2.2. Western Blot Analysis

The total proteins were extracted from ESCC cells (KYSE 30, KYSE 510, and EC9706) using ice-cold RIPA lysis buffer (Thermo Fisher Scientific Inc.). The protein concentration was determined using a bicinchoninic acid protein assay kit. Proteins (20 *μ*g) were heated in loading buffer at 100°C for 10 min and separated by 10% SDS-PAGE, followed by transfer onto PVDF membranes. After blocking with 5% skimmed milk at room temperature for 1 h, the membranes were probed at 4°C overnight with primary antibodies against BCL-2 (1:1000 dilution; product no. D55G8), caspase-3 (1:1000 dilution; product no. D3R6Y), caspase-7 (1:1000 dilution; product no. D2Q3L), caspase-9 (1:1000 dilution; product no. 9502), PARP (1:1000 dilution; product no. 46D11), ATG5 (1:1000 dilution; product no. 2630), BECLIN-1 (1:1000 dilution; product no. 3738), GAPDH (1:1000 dilution; product no. D4C6R), *β*-tubulin (1:1000 dilution; product no. 2128; all from Cell Signaling Technology), and TRPC1 (1:1000 dilution; product no. ab110837). After washing in TBST three times, the blots were incubated with the HRP-conjugated secondary antibodies (anti-rabbit IgG; 1:8000 dilution, product no. 7074; Cell Signaling Technology Inc.) at room temperature for 1 h. Finally, an enhanced chemiluminescence detection system (Imager 600; Amersham; Cytiva) was used to quantify the protein levels with an enhanced chemiluminescent reagent (ECL; Epizyme Inc.).

### 2.3. Cell Counting Kit-8 (CCK-8) Assay

Cell viability was determined using the CCK-8 assay. The KYSE 30, KYSE 510, and EC9706 cells were plated in a 96-well plate at a density of 2000 cells/well. After the cells were attached to the cell plate, SKF96365 was added to each row of cells according to the gradient concentration dilution method and treated for 24, 48, and 72 h, respectively, to detect cell viability. CCK-8 reagent (10 *μ*L; Dojindo Molecular Technologies Inc.) was added to 100 *μ*L RPMI-1640 culture medium at 2 and 3 days, and the sample was incubated in a cell culture incubator for 2 h at 37°C. Absorbance was detected at 450 nm using a microplate reader to generate a cell proliferation curve. At least three determinations were performed in triplicate.

### 2.4. Colony Formation Assay

All cell lines were seeded onto 60-mm plates at a density of 2000 cells/well and repeated three times. After 10 days of incubation, the cells were fixed with 4% paraformaldehyde for 30 min at room temperature and then stained with 0.1% crystal violet solution for 30 min at room temperature. Finally, images were captured, the number of cell colonies was counted, and clones with ≥50 cells were scored as actual colonies. Each cell line was repeated at least three times.

### 2.5. Terminal Deoxynucleotidyl Transferase dUTP Nick End Labeling (TUNEL) Staining

TUNEL is a method for detecting DNA fragmentation by labeling the 3′-hydroxyl termini in the double-strand DNA breaks generated during apoptosis. ESCC cells were cultured in a 96-well microplate for at least 24 h and then induced apoptosis by different SKF96365 concentrations. Then, cells were fixed with 4% paraformaldehyde for 30 min and then cultured with 0.3% TritonX-100 in PBS for 30 min. After washing with PBS three times, the cells were incubated following the instructions of the apoptosis detection kit and stained with 4′,6-diamidino-2-phenylindole (DAPI). Apoptosis was observed and photographed under a fluorescence microscope (Olympus).

### 2.6. Reverse Transcription-Quantitative (RT-q) PCR Analysis

Total RNA was extracted from cells with TRIzol reagent (Thermo Fisher Scientific Inc.) and reverse-transcribed to cDNA using the reverse transcription for PCR Kit (Takara Bio Inc.) according to the standard protocols provided by the manufacturer. qPCR was performed with the ABI7900HT Fast Real-Time PCR system (Applied Biosystems; Thermo Fisher Scientific Inc.) using a SYBR Green PCR Kit (Applied Biosystems; Thermo Fisher Scientific Inc.). The primer sequences used for this paper are presented in Table 1. The following PCR conditions were used: initial denaturation at 95°C for 2 min, followed by 40 cycles of denaturation at 94°C for 15 s, and annealing/extension at 60°C for 1 min. The relative expression of TRPC1, BCL-2, and BECLIN-1 mRNA against GAPDH was calculated using the two methods [14].

### 2.7. Drug and Solution

1-[2-(4-Methoxyphenyl)-2-[3-(4-methoxyphenyl) propoxy] ethyl]imidazole and 1-[*β*-(3-(4-methoxyphenyl)propoxy)-4-methoxyphenethyl]-1H-imidazole hydrochloride (SKF95365) were purchased from Sigma-Aldrich (St. Louis, MO, United States; product no. S7809). To avoid bias caused by different solvents in vivo and in vitro experiments, SKF96365 was dissolved in 0.9% normal saline at a concentration of 50 mM and stored at −80°. Use within the validity period suggested in the manual.

### 2.8. Xenograft Assays

Five-week-old female athymic BALB/c mice were inoculated into the right back with 5 × 10^6^ KYSE 510 cells, respectively. When the tumor size stabilized about 15 days after inoculation (approximately 0.5 cm), animals were randomly assigned to the vehicle and SKF96365 groups. The vehicle group was applied (0.2 mL 0.9% physiological saline), and the SKF96365 group was applied (0.2 mL 20 mg/kg) every other day for 14 days through intraperitoneal injection. At the same time, tumor weight and size were measured regularly. Tumor size was determined by measuring two diameters with electronic vernier calipers according to the authoritative formula: *V* = (*L* × *W*^2^)/2, where *L* is the length and *W* is the width of a xenograft. Within 24 h of the last experiment, the nude mice were anesthetized with 10% lidocaine and then sacrificed by cervical dislocation. After that, the tumors were processed for western blot analysis or immunohistochemical analysis. This animal study was approved by the Institutional Animal Ethics Committee of Zhengzhou University with approval no. ZZU-LAC20220311[05].

### 2.9. Statistical Analysis

All experiments were performed at least three times, and the data were presented as the mean ± standard deviation. In the present study, we followed part of the methodology of Zhu et al. [3, 15]. GraphPad Prism 9.0 was used for statistical analysis. A difference was considered significant when *p* < 0.05.

## 3. Results

### 3.1. SKF96365 Suppresses ESCC Cell Proliferation in a Time- and Dose-Dependent Manner

**Figure 1:**
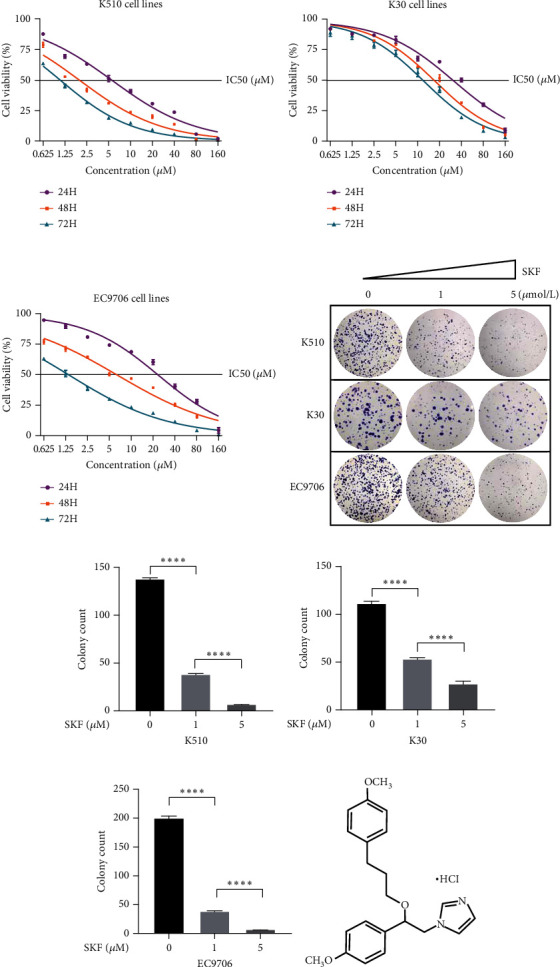
SKF96365 inhibits the proliferation of ESCC cancer cells. (a–c) Inhibitory effect of SKF96365 on K510, K30, and EC9706 cells at different times (24, 48, and 72 h) and different concentrations (0, 0.625, 1.25, 2.5, 5, 10, 20, 40, 80, and 160 *μ*M). (d) Colony formation experiments on ESCC cells. (e–g) Total number of colony formation in different cell lines ((e) K510, (f) K30, and (g) EC9706). (h) Pharmacochemical molecular structure of SKF96365. ^∗∗∗∗^*P* < 0.0001.

Firstly, we detected the inhibitory effect of SKF96365 on several common esophageal cancer cell lines in vitro (Figures 1(a), 1(b), and 1(c)). Experimental results showed that SKF96365 significantly inhibited the growth of tumor cells in a time-dependent and dose-dependent manner. The half maximal inhibitory concentration (IC50) values of K510, K30, and EC9706 after SKF96365 treatment for 24 h were about 5.58, 31.52, and 23.78 *μ*M. And Figures 1(d), 1(e), 1(f), and 1(g) exhibit that colony formation was also significantly inhibited by SKF96365 for 24 h. The chemical structure of SKF96365 is shown in Figure 1(h).

### 3.2. SKF96365 Induces Apoptosis in ESCC Cells

**Figure 2:**
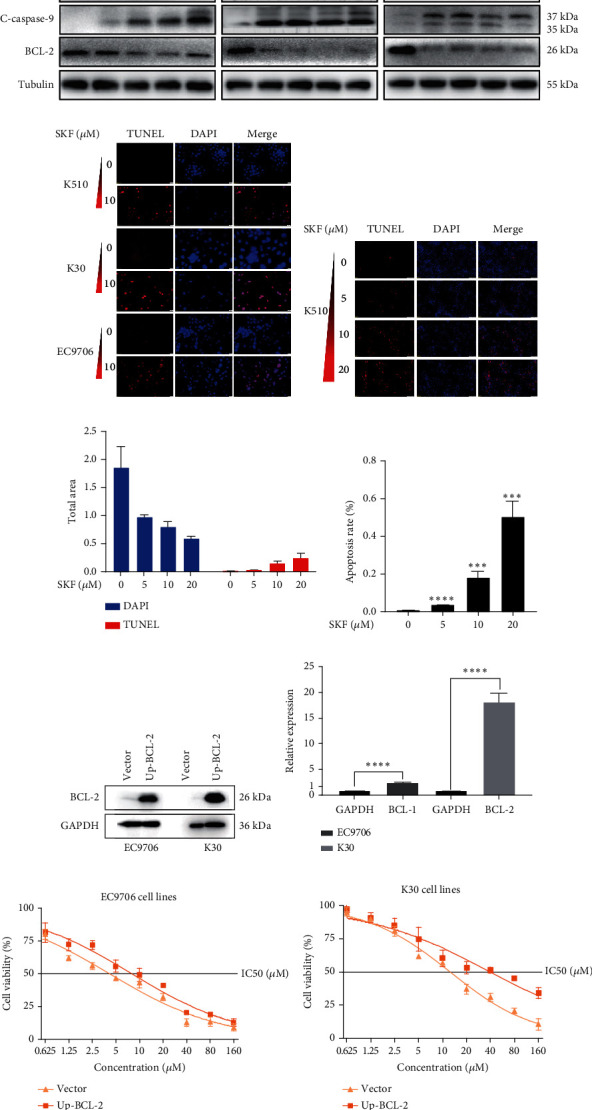
SKF96365 induces apoptosis in ESCC cancer cells. (a) The expression of apoptosis-associated proteins in cells treated with SKF96365 for 24 h. (b) TUNEL staining for K510, K30, and EC9706 cells after being treated with SKF96365 (TUNEL [orange], DAPI [blue], and MERGE [purple]). (c) TUNEL staining for K510 in a concentration gradient and the total (d) apoptosis area and (e) apoptosis rate were calculated. (f, g) WB and q-PCR were used to detect the overexpression of BCL-2 in EC9706 and K30 cells. (h, i) CCK-8 was used to measure the IC50 between the vector and the UP-BCL-2 groups. ^∗∗∗^*p* < 0.001 and ^∗∗∗∗^*p* < 0.0001.

To investigate whether SKF96365-induced cell growth inhibition is dependent on apoptosis, we detected apoptosis-related proteins in SKF96365-treated cells. Western blot analyses showed that cleaved-PARP (C-PARP) and cleaved caspase-9 (C-caspase-9) were increased and BCL-2 was decreased after the treatment of SKF96265 for 24 h. Also, we performed TUNEL assay to detect DNA fragmentation during the apoptosis process. Cells were treated with SKF96365 for 24 h before staining with TUNEL assay.  Figure 2(b) shows that there were almost no apoptotic cells in untreated cells of K510, K30, and EC9706, while almost all the cells treated with SKF96365 at 10 *μ*M showed apoptosis. What is more, Figure 2(c) shows a dose-dependent increase in the apoptosis rate of K510 cells as well as K30 and EC9706 cells (not shown). In order to further verify the effect of drugs on apoptosis, we overexpressed BCL-2 in K30 and EC9706 cell lines. Figures 2(f) and 2(g) showed the successful overexpression of BCL-2 at protein and mRNA levels, respectively. Compared with EC9706, it is obvious that the overexpression of BCL-2 is more significant in the K30 cell line (Figures 2(f) and 2(g)), as well as the enhancement of drug resistance (Figures 2(h) and 2(i)). CCK-8 experiments showed that the cells in the UP-BCL-2 group were more resistant to SKF96365, and the IC50 (48 h) value was also increased, which indicates that SKF96365 can cause apoptosis of esophageal cancer cells.

### 3.3. SKF96265 Suppresses Xenograft Growth in Mice

**Figure 3:**
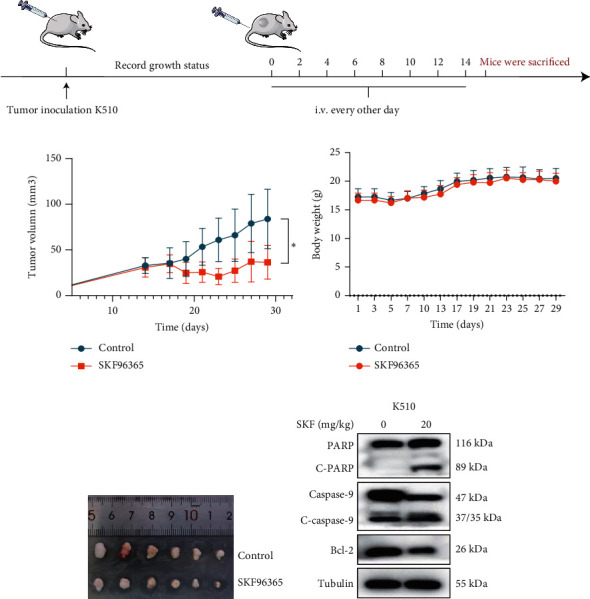
SKF96365 inhibits xenograft growth through apoptosis in vivo. (a) Flow chart of the mouse experiment, and specific process is accessible in materials and methods. Tumor (b) volume and (c) weight in each group. (d) Tumors from nude mice treated with 5% normal saline and SKF96365. (e) Western blot was used to detect the expression of apoptotic proteins including PARP, caspase-9, and BCL-2.

The previous results strongly suggested that SKF96365 could exhibit antitumor activity in vivo. We continued to study the antitumor effect of SKF96365 in a xenograft-bearing mouse model. The K510 cell line was used in the mouse transplant model, and SKF96365 was intraperitoneally injected (control group: 0.2 mL 0.9% physiological saline; SKF96365 group: 0.2 mL 20 mg/kg) every other day for 14 days (Figure 3(a)). The burden of transplanted tumor was estimated by measuring the volume of transplanted tumor in mice. Tumor load was substantially reduced in the SKF96365 group compared to the control group (Figures 3(b), 3(c), and 3(d)). Body weight changes in mice were also recorded during observation to elucidate potential nonspecific toxicity or side effects of SKF96365. It was found that SKF96365 injection did not significantly reduce the body weight of mice compared with the control group (Figure 3(c)). In addition, western blot assay showed that the proapoptotic proteins C-PARP and C-caspase-9 in tissue cells were highly expressed in the SKF96365-treated xenografts, while the antiapoptotic protein BCL-2 was lowly expressed (Figure 3(e)). The results showed that SKF96365 could play an antitumor role by regulating PARP, caspase-9, and BCL-2 apoptosis pathways in vivo.

### 3.4. SKF96365 Downregulates TRPC1 Expression

**Figure 4:**
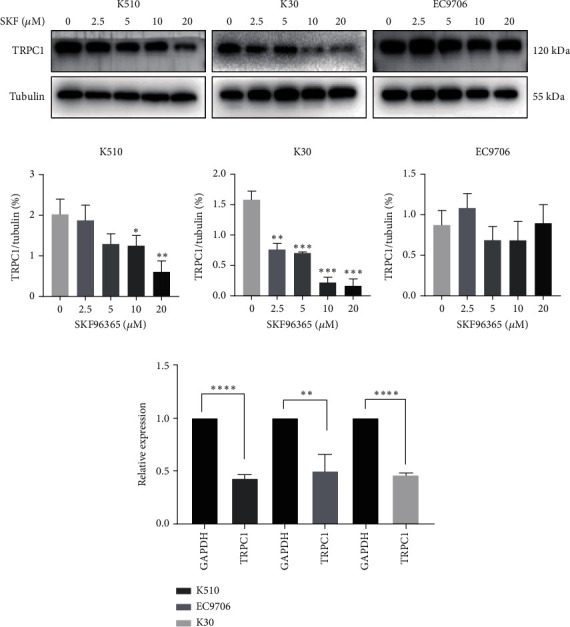
SKF96365 downregulates TRPC1 expression in ESCC cells. (a) Western blot bands of TRPC1 in ESCC cells after being treated with SKF96365 for 24 h. (b–d) Western blot bands were quantified by ImageJ densitometric analysis and normalized by tubulin. (e) Real-time PCR analysis of TRPC1 expression in ESCC cell lines. ^∗^*p* < 0.05, ^∗∗^*p* < 0.01, ^∗∗∗^*p* < 0.001, and ^∗∗∗∗^*p* < 0.0001.

SKF96365 is a typical TRPC inhibitor. To find the calcium channel regulated by SKF96365, proteins and RNAs were extracted 24 h after drug treatment, and their expression levels were detected. Western blot bands and quantification showed that TRPC1 decreased significantly with increasing drug concentration in K510 and K30 cell lines, but no statistical difference was shown in EC9706 (Figures 4(a), 4(b), 4(c), and 4(d)). The mRNA expression of TRPC1 decreased in all three cell lines (Figure 4(e)). These results suggest the possibility of calcium ions regulating cell proliferation and apoptosis.

### 3.5. SKF96365 Alters Autophagy-Related Proteins

**Figure 5:**
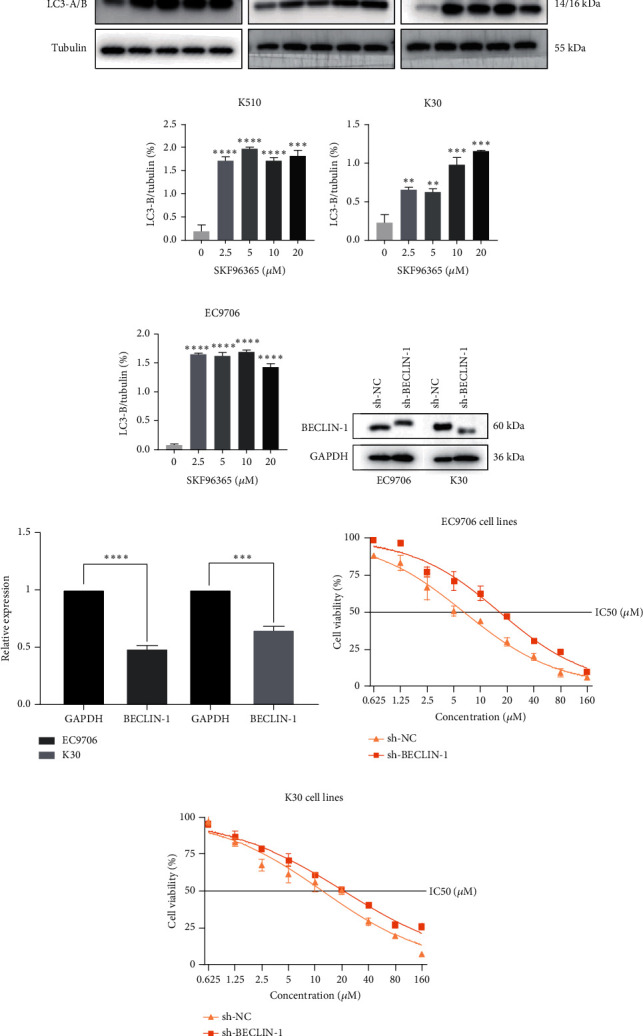
SKF96365 alters autophagy-related proteins. (a) Western blot bands of LC3-A/B in ESCC cells after being treated with SKF96365 for 24 h. (b–d) Western blot bands were quantified by ImageJ densitometric analysis and normalized by tubulin. (e, f) WB and q-PCR were used to detect the downregulated BELIN-1. (g, h) CCK-8 was used to measure the IC50 between the sh-NC and the sh-BECLIN-1 groups. ^∗∗^*p* < 0.01, ^∗∗∗^*p* < 0.001, and ^∗∗∗∗^*p* < 0.0001.

The last literature has shown that SKF96365 can also induce autophagy after altering the expression of TRPC1 through inducing ER stress [16]. And LC3-A/B acts as the key regulator in the autophagy process. Therefore, we examined the expression levels of LC3-A/B by western blot assay (Figure 5(a)). The results (Figures 5(b), 5(c), and 5(d)) revealed that SKF96365 treatment significantly upregulated the ratio of LC3-B/tubulin, hinting at the occurrence of autophagy. BECLIN-1 is another important player in the autophagy formation process, and we attempt to use it to prove the existence of autophagy again. Lentivirus packaging techniques were used to knock down BECLIN-1 expression in EC9706 and K30 cell lines, and Figures 5(e) and 5(f) show that the two cell lines achieved similar knockout effects at protein and mRNA levels. Subsequently, a 48-h CCK-8 assay detected increased drug resistance and an IC50 value in the sh-BECLIN-1 group (Figures 5(g) and 5(h)). All in all, LC3 and BECLIN-1 both demonstrated the autophagy effect of SKF96365.

## 4. Discussion

A growing number of evidences supports the contribution of Ca^2+^ signaling alterations to tumor progression and metastasis, suggesting that targeting calcium influx and downstream signaling driven by calcium influx may hold promise for alternative approaches to cancer therapy [17, 18]. Several calcium channel blockers have been tested in clinical trials [19, 20]. However, the function mechanisms of these drugs are unclear, and there is a lack of basic experiments in vitro and in vivo. Understanding the molecular mechanisms may help improve the efficacy of these agents.

In our study, SKF96365 showed strong growth inhibition against KYSE 30, KYSE 510, and EC9706 cell lines in vitro with increasing time and concentration. Colony formation experiments also support this view (Figures 1(d), 1(e), 1(f), and 1(g)). Then we wondered whether apoptosis had taken place. TUNEL is a more sensitive method for the detection of apoptosis than Annexin V-FITC flow cytometry [21]. ESCC cells were treated with different concentrations of SKF96365 for 24 h and stained with TUNEL kits, and the results showed significant apoptosis in three cell lines. Western blot was also used to explore the mechanism of apoptosis. PARP is a Zn-dependent eukaryotic binding protein that specifically recognizes broken ends of DNA and binds to repair DNA damage [22]. In the early stage of apoptosis, the 113 kDa PARP can be hydrolyzed into 89 and 24 kDa fragments by the caspase family. As shown in Figure 2, the K510 cell line showed significant hydrolyzation of PARP. In addition, the widely recognized mechanism of apoptosis is about the caspase family. Caspase-9 exists in cells in the form of an inactive proenzyme. When cells are stimulated by apoptosis signals, apaf-1 can bind to procaspase-9, making procaspase-9 cleaved and activated. Activated caspase-9 leads to the cleavage of caspase-3 and caspase-7, triggering caspase cascade activation and leading to massive apoptosis of cells [23, 24]. However, after being treated with SKF96365 for 24 h, the spliceosome of caspase-7 was not significantly detected in this experiment. Then, we can see that the antiapoptosis gene BCL-2 showed significant inhibition. As we all know, the expression of BCL-2 is believed to be antiapoptotic and causes drug resistance in many cancer species, including esophageal cancer [25, 26]. And we have verified this point by increasing the IC50 value by overexpressing BCL-2. In fact, there is another mechanism by which apoptotic events occur. Caspase-9 is regulated by BCL-2 and thus triggers apoptotic events rather than acting primarily as the initiator of the caspase cascade [27]. Our evidence clearly supports the latter theory. Subsequently, we demonstrated the antitumor effect of SKF96365 by inducing apoptosis in animal models.

Since SKF96365 is a typical TRPC inhibitor, we speculated that apoptosis might be related to calcium ion regulation. In fact, calcium ions are highly related to apoptotic signaling. Mitochondria and endoplasmic reticulum (ER) pathways are two closely related apoptotic pathways. Under certain stimulation, mitochondria will take up calcium ions released by ER, which is the most important intracellular calcium reservoir. Overload of calcium ions will lead to mitochondrial damage, release of cytochrome C, activation of the caspase family, PARP cleavage and DNA breakage, and finally induce cell apoptosis [28]. In addition, BCL-2 can reduce the concentration of calcium ions in the ER by decreasing the expression of calreticulin and calcium pumps in the ER lumen [29]. BCL-2 also inhibits apoptosis by blocking Ca^2+^ influx and cytochrome C release through the mitochondrial pathway mediated by calcium ions [30]. Therefore, we performed the detection of the expression of TRPC family proteins (TRPC1, TRPC 3, TRPC 5, and TRPC 7) at mRNA and protein levels, respectively, and they collectively showed the downregulation of TRPC1 and a long tail effect.

TRPC1 is both a traditional TRP channel and a voltage-independent ion channel. A large number of literatures have shown that TRPC1 can regulate the development of multiple cancers in calcium-dependent or nondependent forms [31]. Schnipper et al. found that in pancreatic ductal adenocarcinoma, TRPC1 channels form PI3K/CaM complexes to regulate cancer cell proliferation in a calcium-independent manner [32]. However, in most cancers, TRPC1 is associated with intracellular calcium ion regulation, and high expression of TRPC1 is associated with poor prognosis in patients with tumors, such as tongue squamous cell carcinoma, endometrial carcinoma, kidney cell carcinoma, colorectal cancer, breast cancer, stomach cancer, and prostate cancer [33–39]. In esophageal cancer, it has been shown that SKF96365 treatment can significantly enhance intracellular calcium shock levels [40]. Therefore, we believe that SKF96365 plays an antitumor role in esophageal cancer by disturbing the expression of TRPC1. However, one study showed that downregulating TRPC1 expression promoted the development and metastasis of EC9706 cell lines in vitro, which contradicted our results [41]. We hypothesized that the antitumor mechanism of SKF96365 is complex, and the downregulation of TRPC1 is only a triggering point. In addition, studies have shown that overexpression or knockdown of TRPC1 has no function but only functional tetramer structures that form similar voltage-gated calcium channels with TRPC3, TRPC4, and TRPC5 [42–45]. In the future, it is necessary to fully understand the mechanism of SKF96365 inhibiting tumor through calcium channels.

When the TRPC1 gene is silenced or the TRPC1 protein is lowly expressed, calcium influx is reduced and intracellular free Ca^2+^ concentration is decreased, which is in line with the status of autophagy [46]. Additionally, calcium channel blockers induced consistent ER stress, which sets off a protective response mediated at least partly by autophagy [47]. Autophagy and apoptosis can be triggered by SKF96365 in multiple cells at the same time [9, 16, 48, 49]. Therefore, our experiment explored the LC3-B/tubulin ratio of K510, K30, and EC9706 cell lines after 24 h treatment of SKF96365 with different gradient concentrations and found the autophagy to be strongly activated, which may have led to rapid cell death. Furthermore, when we knocked down an important gene (BECLIN-1) in the autophagy process, we found cells were resistant to the treatment of SKF96365. We think this may be because autophagy is involved in the clearance of energy and metabolites, and blocking this process reduces sensitivity to drugs. Recent evidence even suggests that autophagy can delay Ca^2+^ influx after TRPC1 inhibition, thus delaying the process of apoptosis [50]. However, whether autophagy delays the occurrence of apoptosis needs to be further verified by autophagy inhibitors.

In this paper, we regret not providing a comprehensive overview of the mechanism of action of SKF96365 due to constraints, but our findings leave room for further exploration. (1) We found that SKF96365 could induce obvious apoptosis, autophagy, and cycle arrest in three esophageal cancer cell lines (results were not fully shown). Whether protective autophagy and apoptosis promote or inhibit each other is unknown. (2) Whether TRPC1 acts alone or in conjunction with other proteins to form polymers is worth exploring. (3) SKF96365 and cisplatin were used in combination in esophageal cancer cell lines, and the CCK-8 experiment showed that SKF96365 could significantly increase the sensitivity of cisplatin, suggesting the potential value of drug combination treatment.

## 5. Conclusions

In conclusion, our results demonstrate that SKF96365 inhibits tumor growth by inhibiting TRPC1 in esophageal cancer cells and inducing apoptosis (PARP, caspase-9, and BCL-2) and autophagy (LC3-A/B). Hence, our discovery, for the first time, provides further insights into the anticancer potential of TRPC inhibitors in ESCC.

## Figures and Tables

**Table 1:** Primer sequence summary.

**Gene**	**Sequence**
TRPC1	q-F: 5′-AGGATAGCCTCCGGCATTC-3′
	q-R: 5′-TTCCACCTCCACAAGACTTAGT-3′
GAPDH	q-F: 5′-GCTGAACGGGAAGCTCACTG-3′
	q-R: 5′-GTGCTCAGTGTAGCCCAGGA-3′
BCL-2	q-F: AGATGTCCAGCCAGCTGCAC
	q-R: TGTTGACTTCACTTGTGGCC
BECLIN-1	q-F: GGCTGAGAGACTGGATCAGG
	q-R: CTGCGTCTGGGCATAACG

Abbreviations: F, forward; q, qPCR; R, reverse.

## Data Availability

The raw data used to support the findings of this study are available from the corresponding authors upon request.

## Nomenclature

BCL-2: B-cell lymphoma-2
CCK-8: Cell Counting Kit-8
ER: endoplasmic reticulum
ESCC: esophageal squamous cell carcinoma
PARP: poly(adenosine diphosphate–ribose) polymerase
PCR: polymerase chain reaction
SOCE: store-operated calcium entry
TRPC: transient receptor potential canonical
